# Misfolding, altered membrane distributions, and the unfolded protein response contribute to pathogenicity differences in Na,K-ATPase *ATP1A3* mutations

**DOI:** 10.1074/jbc.RA120.015271

**Published:** 2020-11-22

**Authors:** Elena Arystarkhova, Laurie J. Ozelius, Allison Brashear, Kathleen J. Sweadner

**Affiliations:** 1 Laboratory of Membrane Biology, Department of Neurosurgery, Massachusetts General Hospital, Boston, Massachusetts, USA; 2 Department of Neurology, Massachusetts General Hospital, Boston, Massachusetts, USA; 3 Department of Medicine, University of California at Davis Medical School, Sacramento, California, USA

**Keywords:** Na^+^/K^+^-ATPase, genetic disease, protein misfolding, unfolded protein response (UPR), N-linked glycosylation, endoplasmic reticulum stress (ER stress), endoplasmic reticulum associated protein degradation (ERAD), subcellular fractionation, eukaryotic initiation factor 2alpha (eIF2 α), 4-phenylbutyrate (4PBA), 4PBA, 4-phenylbutyrate, also known clinically as Buphenyl, BAD, Bcl-2 agonist of cell death, eIF2α, eukaryotic initiation factor 2α, ER, endoplasmic reticulum, ERAD, ER-associated degradation, IRE1α, serine/threonine-protein kinase/endoribonuclease inositol-requiring enzyme 1α, a dual-function transmembrane ER receptor, SDS, sodium dodecyl sulfate, tet, tetracycline, UPR, unfolded protein response, XBP1s, X-box binding protein 1, spliced form, a transcription factor

## Abstract

Missense mutations in *ATP1A3*, the α3 isoform of Na,K-ATPase, cause neurological phenotypes that differ greatly in symptoms and severity. A mechanistic basis for differences is lacking, but reduction of activity alone cannot explain them. Isogenic cell lines with endogenous α1 and inducible exogenous α3 were constructed to compare mutation properties. Na,K-ATPase is made in the endoplasmic reticulum (ER), but the glycan-free catalytic α subunit complexes with glycosylated β subunit in the ER to proceed through Golgi and post-Golgi trafficking. We previously observed classic evidence of protein misfolding in mutations with severe phenotypes: differences in ER retention of endogenous β1 subunit, impaired trafficking of α3, and cytopathology, suggesting that they misfold during biosynthesis. Here we tested two mutations associated with different phenotypes: D923N, which has a median age of onset of hypotonia or dystonia at 3 years, and L924P, with severe infantile epilepsy and profound impairment. Misfolding during biosynthesis in the ER activates the unfolded protein response, a multiarmed program that enhances protein folding capacity, and if that fails, triggers apoptosis. L924P showed more nascent protein retention in ER than D923N; more ER-associated degradation of α3 (ERAD); larger differences in Na,K-ATPase subunit distributions among subcellular fractions; and greater inactivation of eIF2α, a major defensive step of the unfolded protein response. In L924P there was also altered subcellular distribution of endogenous α1 subunit, analogous to a dominant negative effect. Both mutations showed pro-apoptotic sensitization by reduced phosphorylation of BAD. Encouragingly, however, 4-phenylbutyrate, a pharmacological corrector, reduced L924P ER retention, increased α3 expression, and restored morphology.

Heterozygous mutations in the genes for three Na,K-ATPase catalytic subunit isoforms can result in debilitating neurologic syndromes, but the presentations vary widely in severity. The mildest syndromes have onset in adults or youth and are often inherited: axonal Charcot-Marie-Tooth neuropathy (CMT2) in *ATP1A1* (1), familial hemiplegic migraine (FHM2), epilepsy, or both in *ATP1A2* (2), and rapid-onset dystonia-parkinsonism (frequently triggered by stress) in *ATP1A3* (3). In *ATP1A3* there are also three fever-induced syndromes, typically with childhood onset: relapsing encephalopathy with cerebellar ataxia (4), fever-induced paroxysmal weakness and encephalopathy (5), and cerebellar ataxia, areflexia, pes cavus, optic nerve atrophy, and sensorineural deafness (6). More severe syndromes have onset in infancy. First, alternating hemiplegia of childhood has onset from 0 to 18 months of age, characterized by recurring paroxysmal symptoms, and often seizures. Its mutations are usually in *ATP1A3*, rarely in *ATP1A2* (7, 8). Certain other mutations produce even worse outcomes in infancy: early infantile epileptic encephalopathy with profound developmental delay and sometimes hypotonia or microcephaly (cases in all three genes (9, 10, 11)).

Defects in pump function have been documented in many Na,K-ATPase mutations, including lack of detectable activity; reduced affinity for Na^+^ and sometimes for K^+^; changes in the balance between conformations; changes in voltage sensitivity; loss of a normal inward proton current; or abnormal cation permeabilities (2, 7, 9, 12, 13, 14). Any of these changes in *ATP1A3* should impact neuronal electrophysiology *via* their effects on ion distributions and membrane potential. However, a salient genetic feature is that, with rare exceptions, disease-causing mutations in the Na,K-ATPase subfamily of the P-type ATPases (ATPases that employ transient auto-phosphorylation of an aspartate in the active site) are missense and heterozygous, whereas truncation or recessive mutations are lacking. This strongly suggests that, for symptom manifestation in the syndromes we know about, reduced pump activity is not sufficient: the damaged protein must be present.

The question is why some mutations cause profoundly worse syndromes. For rigorous comparisons, we stably introduced mutated human *ATP1A3* in tetracycline-inducible, isogenic HEK-293 cell lines at a fixed FRT recombination site (Flp-In). In this system, the levels of wildtype (WT) exogenous α3 and endogenous α1 proteins were found to be comparable, and they competed with one another for expression (15). The endogenous α1 is sensitive to the inhibitor ouabain, whereas the introduced α3 was given two benign mutations that reduce its affinity ∼3 orders of magnitude. Eleven different mutations were tested initially, and notably, the severity of the human phenotype did not correlate with whether there was enough residual α3 activity at the membrane to keep the cells alive after inhibition of endogenous α1 with ouabain (15).

Oddly, D366H, a mutation of the aspartate in the active site that is indispensable for hydrolyzing ATP, and that did not support cell growth, nonetheless produced only the mildest syndrome, rapid-onset dystonia-parkinsonism. In contrast, *ATP1A3* mutation L924P, now found in two severe cases of early infantile epileptic encephalopathy (15, 16), had partial impairment of biosynthesis and trafficking but enough activity in the plasma membrane to support cell growth in culture. D923N- and L924P-expressing cells supported cell growth but differed in Na,K-ATPase trafficking and the aggregation of misfolded protein, as did two inactive mutations, D743H and D742Y (15). This led to the hypothesis that greater pathogenicity was due to cellular consequences of misfolding in addition to impaired function.

The Na,K-ATPase is an abundant membrane protein in brain. Consequently, its misfolding during biosynthesis could have a cell-intrinsic impact through the unfolded protein response (UPR), which surveys protein misfolding in the ER and mounts a multifaceted response (17). Initially the UPR is defensive, expanding the protein folding capacity of the cell, but if misfolded protein accumulates persistently, the UPR proceeds to apoptosis. The hypothesis tested here was that Na,K-ATPase mutations differing in patient outcome also differ in misfolding and the extent of the UPR response.

Most of the literature on the UPR is focused on acute events triggered by chemicals that strongly disrupt ER function; relatively little work has been done on single misfolded proteins or under chronic conditions (18, 19, 20). This work focuses on two Na,K-ATPase mutations that show classic evidence of misfolding but that have adapted successfully to long-term culture (15). This makes it possible to test how the adaptations differ between mutations and whether they correlate with the severity of human phenotypes.

To understand the complexity of the consequences of α mutations that misfold, it is important to recognize the essential interaction of the α and β subunits. Most membrane proteins have N-linked glycosylation that is used by the ER to monitor progress of the protein’s folding and to control passage to the Golgi apparatus and beyond, for example, CFTR in the well-studied genetic disease cystic fibrosis (21). The Na,K-ATPase β subunit plays this role in the biosynthesis of α subunit (22, 23). The catalytic α and glycoprotein β subunits associate in endoplasmic reticulum (ER) (24), and nascent α subunits compete to cap the total α (15). The glycan in the ER is in the immature high-mannose form, where the cyclical addition and trimming of glucose units controls its interaction with lectin-like chaperones such as calnexin and calreticulin (25), which in turn retain the incompletely folded complex in the ER for further attempts to fold. The ER and mature populations of Na,K-ATPase β subunit can be distinguished by differences in SDS gel mobility caused by immature (high mannose) and mature (multiantennary and sialylated) glycosylation (26). Consequently, the biosynthesis and trafficking of the β subunit is an informative feature of *ATP1A3* α mutations. Here we compared β subunit retention, Na,K-ATPase activity, distribution in cellular membrane fractions, ERAD, and the UPR in WT α3 and two mutations that produce different phenotypes.

## Results

### Retention of Na,K-ATPase β subunit

**Figure 1:**
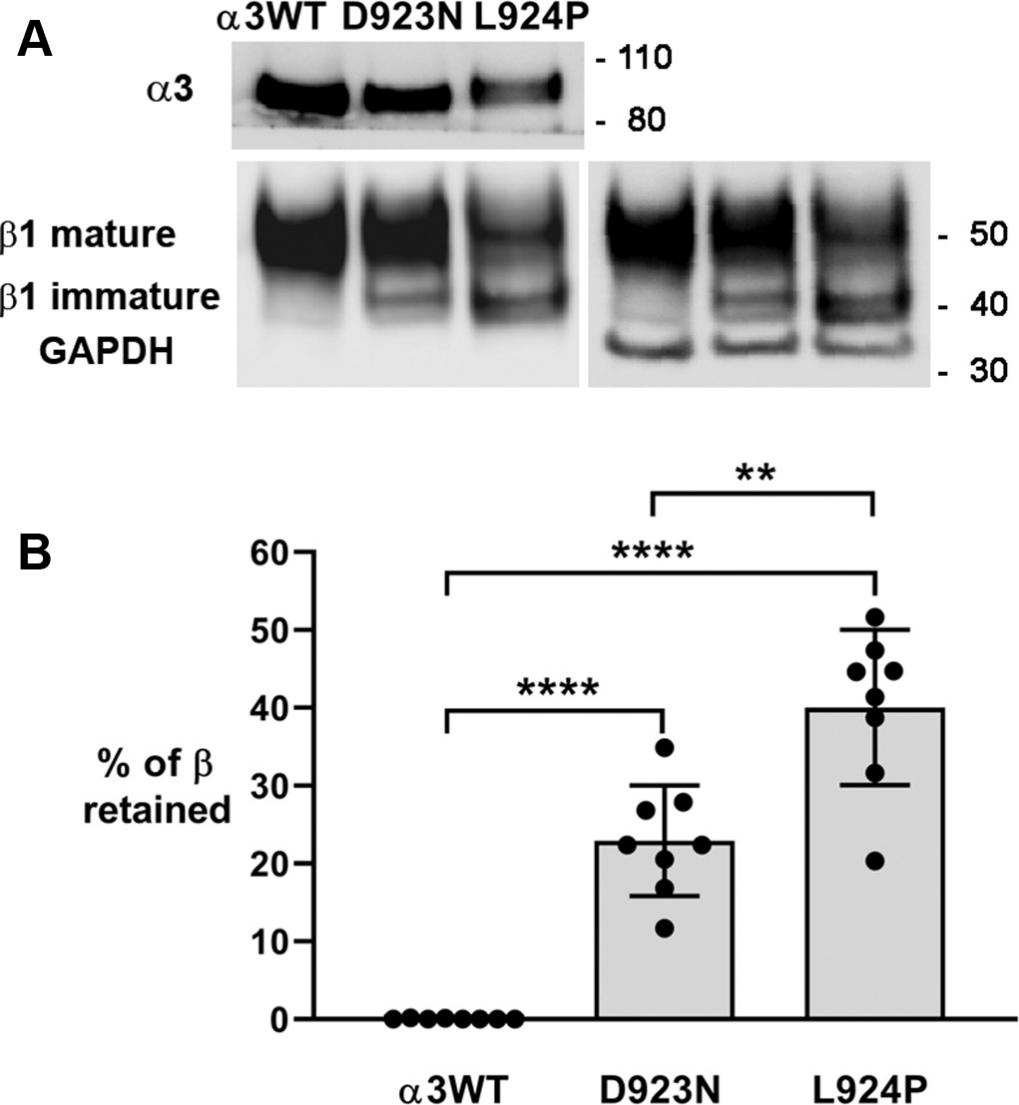
**Retention of Na,K-ATPase β1 subunit in endoplasmic reticulum.** The high-mannose form of Na,K-ATPase β is known to migrate faster, and mature glycosylation is a marker of its progress through the Golgi apparatus. *A*, sections of a single representative Western blot stained with isoform-specific antibodies for α3 (F1, Santa Cruz) and β1 (M17-P5-F11, W. J. Ball Jr, University of Cincinnati), then restained for GAPDH as a loading control. The band for β1 with mature glycosylation was always broad and frequently showed substructures presumably representing differences in glycosylation. *B*, quantification of imager scans of β subunit in n = 9 (WT) or n = 8 (D923N, L924P) independent experiments, with actin or GAPDH as a loading control. What is shown is the fraction of immature β out of the total, which is independent of expression levels. Significance was calculated by two-tailed *t* test. ∗∗*p* < 10^−3^; ∗∗∗∗*p* < 0.0001.

ER retention of β subunit was first detected in two *ATP1A3* mutations with severe phenotypes, L924P and D742Y (15). The first objective here was to determine if retention could also be detected in a milder mutation. Isogenic cells expressing exogenous α3WT, D923N, and L924P were grown with constant tetracycline (tet) induction for at least five passages, and lysates were resolved on Western blots for detection with anti-α3 and anti-β1 antibodies (Fig. 1*A*). The shift of glycosylation from immature to mature forms was used as a marker of ER to Golgi transfer. On SDS gels the β subunit with immature glycosylation runs at 41 kDa, slower than the apoprotein at 32 kDa (usually not detected) but faster than intermediate and mature glycoforms at ∼55 kDa that are generated in the Golgi and trafficked to the plasma membrane (27, 28). The α3WT-expressing cells had little immature β1, presumably representing ongoing biosynthesis. D923N-expressing cells had a higher level of the immature form, whereas L924P-expressing cells had a substantially higher level. The immature form is normally short lived, and its elevation is consistent with a major strategy of the UPR: chaperone-mediated retention in the ER for repeated attempts to fold a protein that is prone to misfolding. When quantified from eight independent experiments, the proportion of β subunit retained was barely detectable in isogenic cells expressing WT α3 (α3WT) but highly significant in both L924P and D923N, albeit lower in D923N, the mutation with the milder phenotype (Fig. 1*B*).

### Fractionation of membranes shows altered trafficking

To further characterize differences in the trafficking of the α and β subunits, sucrose density step gradients were used to collect high-, intermediate-, and low-density membrane fractions. Post-nuclear homogenates of cultures were layered in the middle of step gradients of sucrose density, followed by centrifugation to equilibrium (29). Prior work using fractionated CHO and Sf9 cells interpreted the resulting fractions as enriched in ER, Golgi, and plasma membrane, respectively, using α-glucosidase, α-mannosidase, and alkaline phosphatase activities as markers (30, 31). We used Na,K-ATPase as a plasma membrane marker, GM130 (130 kDa *cis*-Golgi matrix protein, golgin A2) as a Golgi marker, and two ER markers, PERK (PKR-like ER kinase) and PDI (protein disulfide isomerase). Using α3WT, the enrichments were as follows. The dense fraction had 3.5% of Na,K-ATPase, 13% of GM130, 42% of PERK, and 35% of PDI. The intermediate fraction had 3.2% of Na,K-ATPase, 7% of GM130, 11.3% of PERK, and 15.6% of PDI. The light fraction had 93% of Na,K-ATPase, 80% of GM130, 46.7% of PERK, and 49.9% of PDI. The interpretation was that the dense fraction is enriched for rough ER (RER); the intermediate fraction has 3% to 15% of all markers; and the light fraction is enriched for plasma membrane, smooth ER (SER), and much of the Golgi.

**Figure 2:**
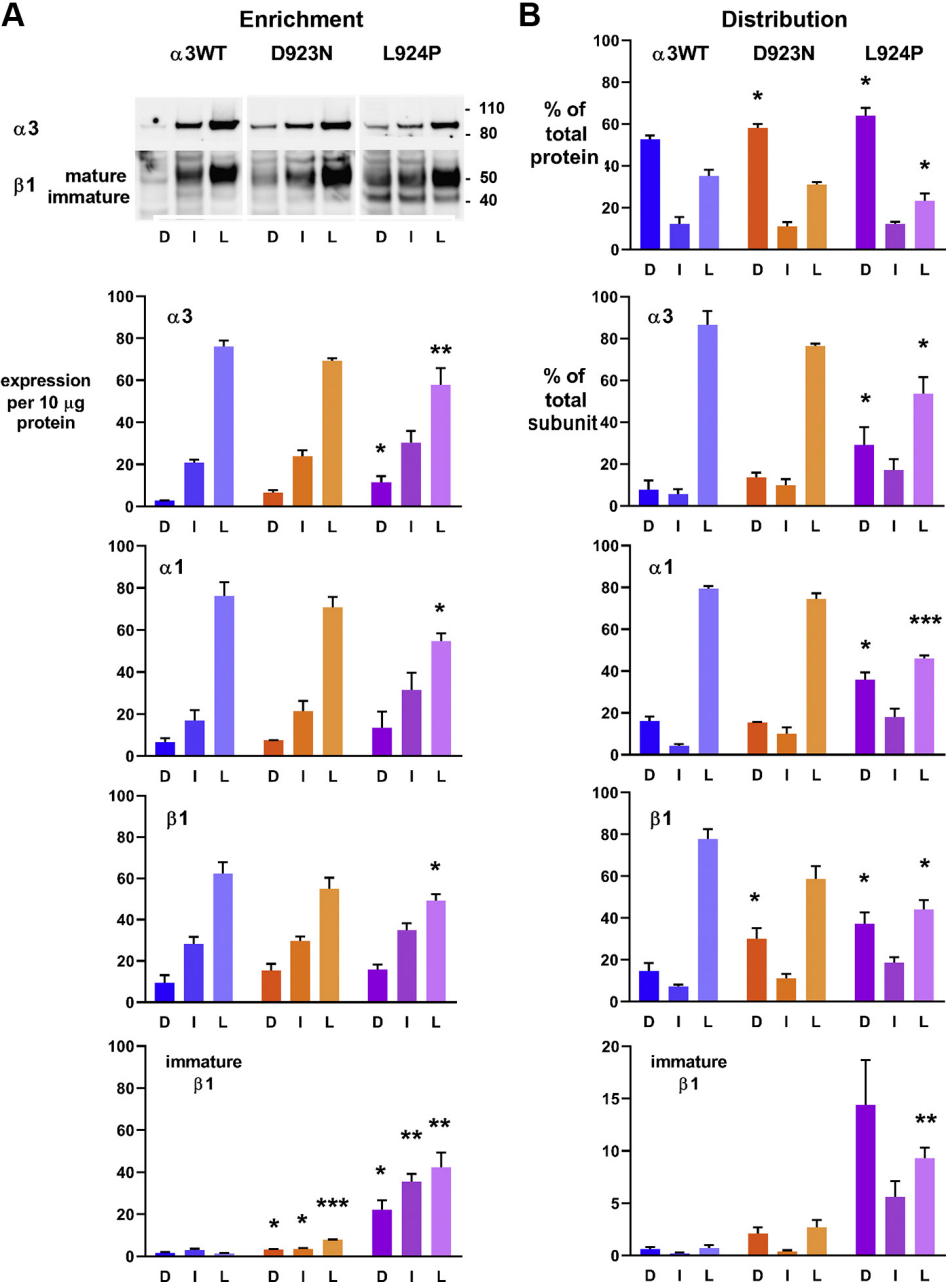
**The subunits of Na,K-ATPase separated on equilibrium density step gradients were analyzed in two ways: by their *enrichment* (relative purity) in dense (D), intermediate (I), and light (L) fractions, and by *distribution* in those fractions, taking into account the total amount of protein in each fraction.** For the Western blot (one representative immunoblot cut and stained with different antibodies), equal protein loading was used to quantify α3, α1, and β1. *A*, enrichment in the light fractions was reduced in both mutations, quantitatively more in L924P. The graphs below the blot show quantification from 3 (α3WT and D923N) or 4 (L924P) independent experiments. Blot intensities from different experiments and antibodies were normalized as described in Experimental procedures. The statistical significance of differences between fractions, and between cell lines, was assessed by *t* test independently. First, all differences between dense and light fractions for each set of data were significant at *p* < 0.05 except for immature β1 in α3WT, where signals were too faint for accurate background subtraction. This dense *versus* light significance is not marked on the graph. Second, among α3, α1, total β1, and immature β1, the significant differences between a mutation and WT at corresponding sucrose densities are marked with asterisks, (∗) *p* < 0.05, (∗∗) *p* < 0.005, and (∗∗∗) *p* < 0.0005. For D923N, only the immature β1 differences were significant. Notice that, for α3, there appears to be more in the mutant dense fraction than in WT. In terms of enrichment of light compared with dense fractions, there was an average 18-fold enrichment in plasma membrane for α3WT, but only 10.4-fold for D923N and 7.4-fold for L924P. For β subunit in the same fractions, the enrichment was only 6.5-fold for α3WT and 3.6- to 3.7-fold for both mutations. *B*, distribution of total protein and each subunit in the same gradients was compared. Statistical significance was tested for the distributions for mutants compared with α3WT. There was a statistically significant shift in the distribution of total protein to the dense fraction in D923N and L924P cells and a decrease in the amount distributed to the light fraction in L924P cells, consistent with remodeling of the intracellular membranes. The extent of redistribution of α3 is consistent with the premise that there is an adaptive UPR response elicited by L924P. It was unexpected that α1 and β1 distributions were equally affected.

These enrichments were sufficient to detect changes in membranes and Na,K-ATPase subunits in the adapted mutation-expressing cells (Fig. 2). For these experiments, cells were grown chronically in both tetracycline and ouabain, conditions in which the cells depend on the activity of the ouabain-resistant α3 in the plasma membrane. Figure 2 shows enrichment (levels per milligram protein of α3, α1, and β1 subunits) and distribution (totals per fraction) for each cell line. Figure 2*A* has a representative Western blot with equal loading of protein and the quantification of replicate experiments. It can be seen that α3 in L924P was significantly less enriched than the α3WT in the light fraction and more in the dense fraction, consistent with retention in RER. Unexpectedly, α1 and β1 were also less enriched in the light fractions, and the enrichment for β subunit appeared to be consistently skewed toward higher densities. This raises the possibility that there may be an excess of β that is not transported by α. Immature β subunit was clearly not confined to the RER fraction when it was present. Since it was not detected at the surface (15), in the lighter fractions it presumably was in the SER.

Figure 2*B* looks at distribution of the Na,K-ATPase rather than its purity; multiplying by the protein in each fraction gives the total of each Na,K-ATPase subunit as percentage of the total. This gives a clearer perspective on the differences and better statistical significance. The top graph shows changes in the distribution of total protein. Relative to α3WT cells, the L924P mutant cells showed significant increases in total dense fraction protein (from 53% to 64% of the total) relative to light fraction protein (from 35% to 23% of the total). Absent an apparent change in cell size and surface area, this is consistent with expansion of the RER and underestimation of SER and plasma membrane (32). D923N cells showed a smaller but significant increase in protein in the dense fraction, but the decrease in the light fraction did not reach significance. For α3 subunit, in WT there was a large majority, 86%, of the total in the light fraction. D923N cells had 76% of α3 in the light fraction and more in dense and intermediate ones, consistent with the data of Figure 2*A*. L924P exhibited only 54% of α3 in the light fraction and retained 29% in dense. As also hinted in Figure 2*A*, an unexpected observation was that the distribution of α1 subunit, the endogenous subunit expressed by HEK 293 cells, was just as abnormal as α3 in the L924P mutant cells. This will be discussed below.

The distribution of β1 subunit among the fractions also changed. Total β1 (mature and immature scanned together) showed a graded shift toward denser fractions in both mutations compared with α3WT. The columns marked with asterisks were significant relative to α3WT. Immature β subunit is shown as a percentage of the total β subunit in each fraction. In L924P there was substantial immature β in all three fractions. The percentages of β retained were somewhat lower than that shown in Figure 1 with unfractionated samples, but these differences were not statistically significant. Taken together, the differences revealed by membrane fractionation are consistent with cellular remodeling due to the UPR.

### Residual ATPase activity

Light membrane fractions enriched in Na,K-ATPase were used to measure ouabain-sensitive hydrolysis of ATP to estimate the loss of enzymatic activity in the mutants. Average ouabain-sensitive specific activities were 12.0 μmol/h/mg protein for α3WT; 2.8 for D923N; and 3.6 for L924P. When these values were corrected for the measured total yields of protein in the light membrane fractions (Fig. 2*B*), the recoveries of activity were 100% for α3WT, 20.7% for D923N, and 19.3% for L924P. When normalized to the amount of α3 on blots, D923N was expressed as well as WT and had 80% loss of activity, whereas L924P had a 40% loss of activity but lower expression, consistent with Figure 2*A*. The residual activity explains why cells survive when the endogenous α1 is inhibited with a low concentration of ouabain. The similarity in net activity is consistent with the similar growth curves shown by the two mutant cell lines, where both grew normally except for a 1-day lag in recovery from passaging when grown in ouabain to inhibit endogenous α1 (15). This ATPase assay reports the level of enzymatic competence under V_max_ conditions *in vitro*. A caveat is that separation by density does not ensure that all of the activity found in the light fraction is actually at the surface. Whether any α3 retained in the denser gradient fractions had activity could not be measured because of low enrichment.

The ATPase activity was measured on light membrane fractions that had been frozen and thawed, but detergent was not added to open sealed vesicles. In fact, when the assay was performed with the bovine serum albumin–buffered SDS-stimulation method (33), activities were not significantly changed for α3WT or D923N cells, but activity was completely lost for L924P cells, implying a greater sensitivity to detergent denaturation, *i.e.*, structural lability (data not shown).

### UPR: mutations activate both defensive and proapoptotic signaling

**Figure 3:**
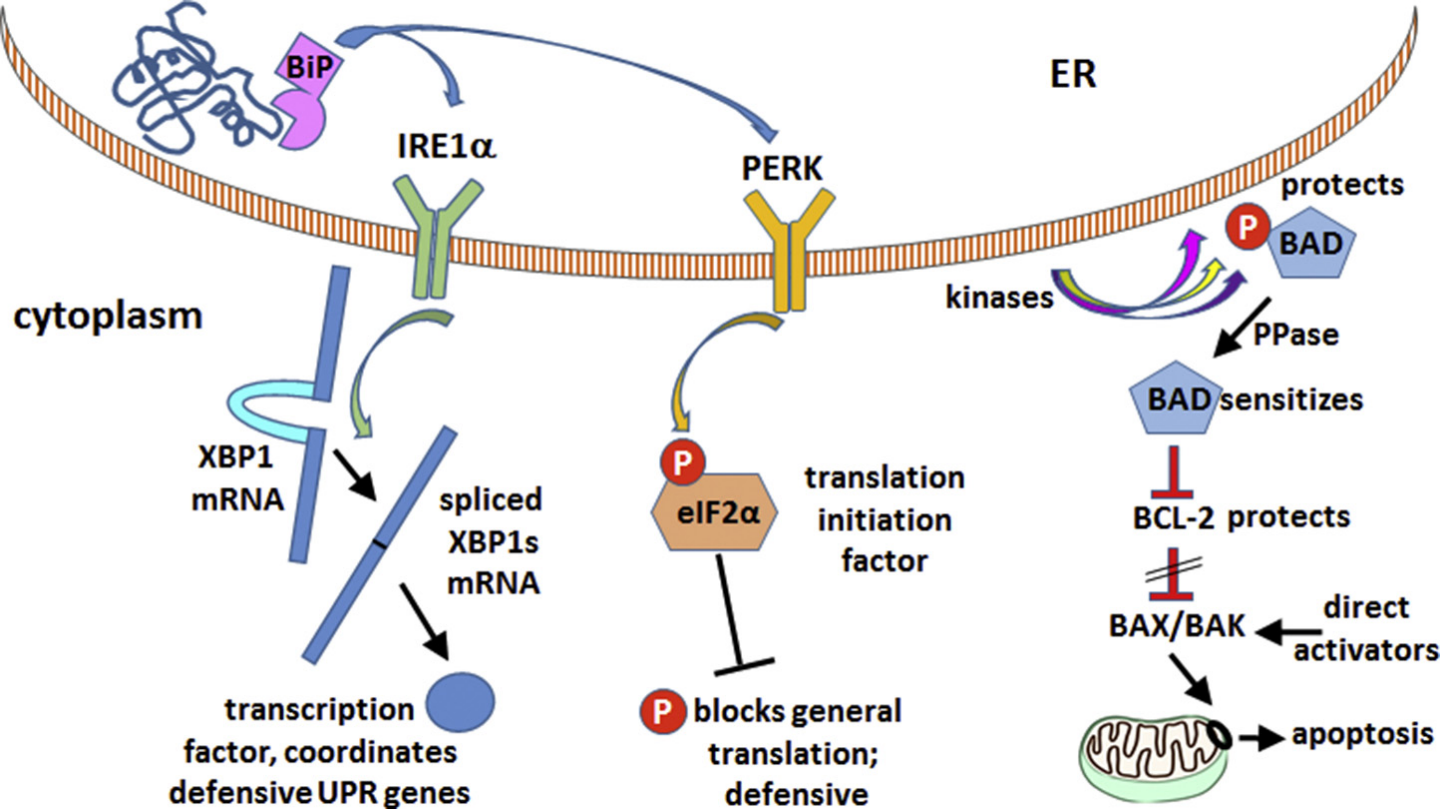
**Relevant pathways of the unfolded protein response (UPR).** This diagram shows only a few of the components of the UPR but highlights three central elements that were examined here. The UPR begins with the recognition of luminal misfolded proteins by the chaperone BiP (GRP78), known to interact with both α and β Na,K-ATPase subunits during biosynthesis in *Xenopus* oocytes (22). The UPR initially activates defensive programs to expand the folding capacity of the ER. (*Left*) Activated IRE1α has a cytoplasmic nuclease activity needed to splice the XPB1 mRNA to XBP1s, changing its reading frame so that it encodes a master transcription factor for the defensive arm of the UPR. (*Middle*) Activated PERK phosphorylates an essential translation initiation factor, eIF2α. This attenuates translation, reducing the stress on the ER and making it possible for its resources to be redirected to defensive adaptations (37). (*Right*) If aggregated proteins nonetheless accumulate, the UPR activates apoptosis (38). The pathway utilizes the BCL-2 family proteins that regulate the formation of mitochondrial pores (*black circle*) by BAX and BAK to release cytochrome c (38). Current models hold that BAX and BAK are activated directly by members of one arm of the BCL-2 family (the direct activators), in response to various signals. Apoptosis is constitutively restrained, however, by BCL-2 itself, which binds and blocks BAX and BAK. For apoptosis to proceed, BCL-2 needs to be sequestered (*crosshatch*) by binding to BAD or other members of the sensitizer arm of the BCL-2 family. BAD binding to BCL-2 is attenuated by phosphorylation by a variety of prosurvival kinases (39, 40). Dephosphorylation of BAD by protein phosphatase (PPase), for example, by calcineurin, the Ca^2+^-activated phosphatase, will activate proapoptotic signaling (41). In sum, dephosphorylation of BAD, at Ser99 in this case, is an indication that BAD is free to inactivate BCL-2, making it more likely that BAX and BAK will respond to direct activators, *i.e.*, sensitizing the cell to apoptosis.

The immature glycosylation of β subunit, the retention of α3 and β1 in the dense fraction enriched in ER, and the altered distribution of total protein all are suggestive of activation of the UPR, but more direct evidence is needed. Figure 3 diagrams the ER stress signaling pathways that were investigated.

**Figure 4:**
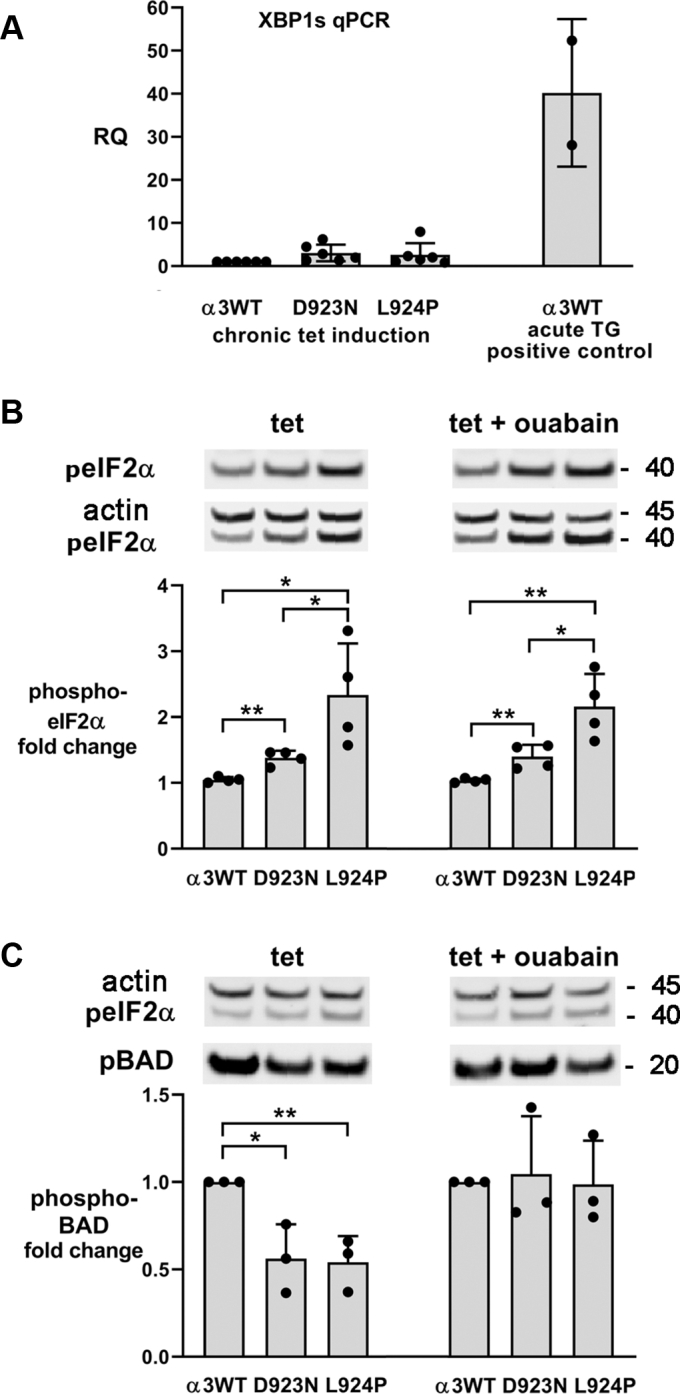
**Unfolded protein response signaling responses to mutation.***A*, the spliced mRNA of the transcription factor XBP1s is an early unfolded protein response marker. Although 5 h of thapsigargin treatment (TG) gave a robust response, the apparent twofold increase in the spliced mRNA with chronic tet induction when compared with α3WT grown without tet or between α3WT and either of the mutation-expressing cells was not statistically significant. RQ stands for relative quantification and is the fold-change relative to the calibrator (actin), 2^-ddCt^. The data are means ± SEM for 4, 6, 5, and 2 experiments. *B*, eIF2α phosphorylation was measured in lysates of cultures that were grown chronically in tet, or in tet + ouabain to inhibit endogenous Na,K-ATPase α1. The representative blot was stained first for phospho-eIF2α (*top*) and then stained again for actin as a loading control. Both images are shown. *C*, BAD phosphorylation measured in the same samples. The blot shown was the same as in B including the eIF2α and actin stain, but BAD runs at lower molecular weight and was stained after phospho-eIF2α and actin. The graphs for both B and C show the means ± SEM from four independent experiments. ∗*p* < 0.01, ∗∗*p* < 0.001, ∗∗∗*p* < 0.0001. Ouabain appeared to protect the cells from dephosphorylation of BAD.

We first looked for mRNA splicing of the transcription factor XBP1, an initial step catalyzed by one of the primary membrane-resident ER stress sensors, IRE1α, which has dual cytoplasmic activities as a kinase and an endoribonuclease required for its splicing (Fig. 4*A*) (18). The spliced mRNA XBP1s encodes a transcription factor that translocates to the nucleus and coordinates the activation of defensive UPR genes. However, XBP1s production is transient (34) and in chronic conditions IRE1α activates later UPR signaling pathways including mRNA degradation (35). We were unable to document statistical significance of a small increase in XBP1s in chronically tet-induced D923N and L924P cell lines, whereas 5 h of thapsigargin treatment (a dependable pharmacological way to activate the UPR) produced 25- to 50-fold increases in hours as a positive control.

We then investigated the phosphorylation of a protein known to play a sustained role in the defensive pathway of the UPR, eIF2α. It is a general translation initiation factor that is inhibited when phosphorylated by PERK, another ER-resident stress sensor (Fig. 3). Inhibition of eIF2α limits the synthesis of total protein and thus reduces the workload for the ER (36). Although total protein synthesis is dialed back, proteins needed for the UPR’s adaptive responses have specialized eIF2α-independent mechanisms of translation initiation, enabling biosynthesis of proteins needed for expansion of the ER and its capacity (37). As shown in Figure 4*B*, eIF2α phosphorylation was consistently increased, with a significantly larger response in L924P cells than in D923N cells. eIF2α levels were unchanged (not shown). Two growth conditions were investigated: tet induction alone, where the exogenous α3 and endogenous α1 are both active, and tet with enough ouabain to inhibit α1, in which case the cells depend on the residual activity of α3. The response of eIF2α was the same in the two conditions.

When defensive responses are insufficient, the UPR leads to apoptosis, and BCL-2 family proteins mediate it. BAD plays a central role as a regulator subject to regulatory phosphorylation from multiple pathways (38, 39, 40, 41). As above, cultures were grown in either chronic tet induction or tet plus ouabain to inhibit α1. There was significant reduction of BAD phosphorylation by almost 50% in both the D923N and L924P cell lines (Fig. 4*C* left). Interestingly, cells grown in both tet and ouabain showed no consistent reduction in BAD phosphorylation (Fig. 4*C* right). There was also no consistent increase or decrease in the level of phospho-BAD in α3WT with ouabain. The apparent protective effect of ouabain will require further investigation, but other unrelated protective effects of ouabain mediated by signaling have been documented (42).

### Rescue of misfolded mutants

**Figure 5:**
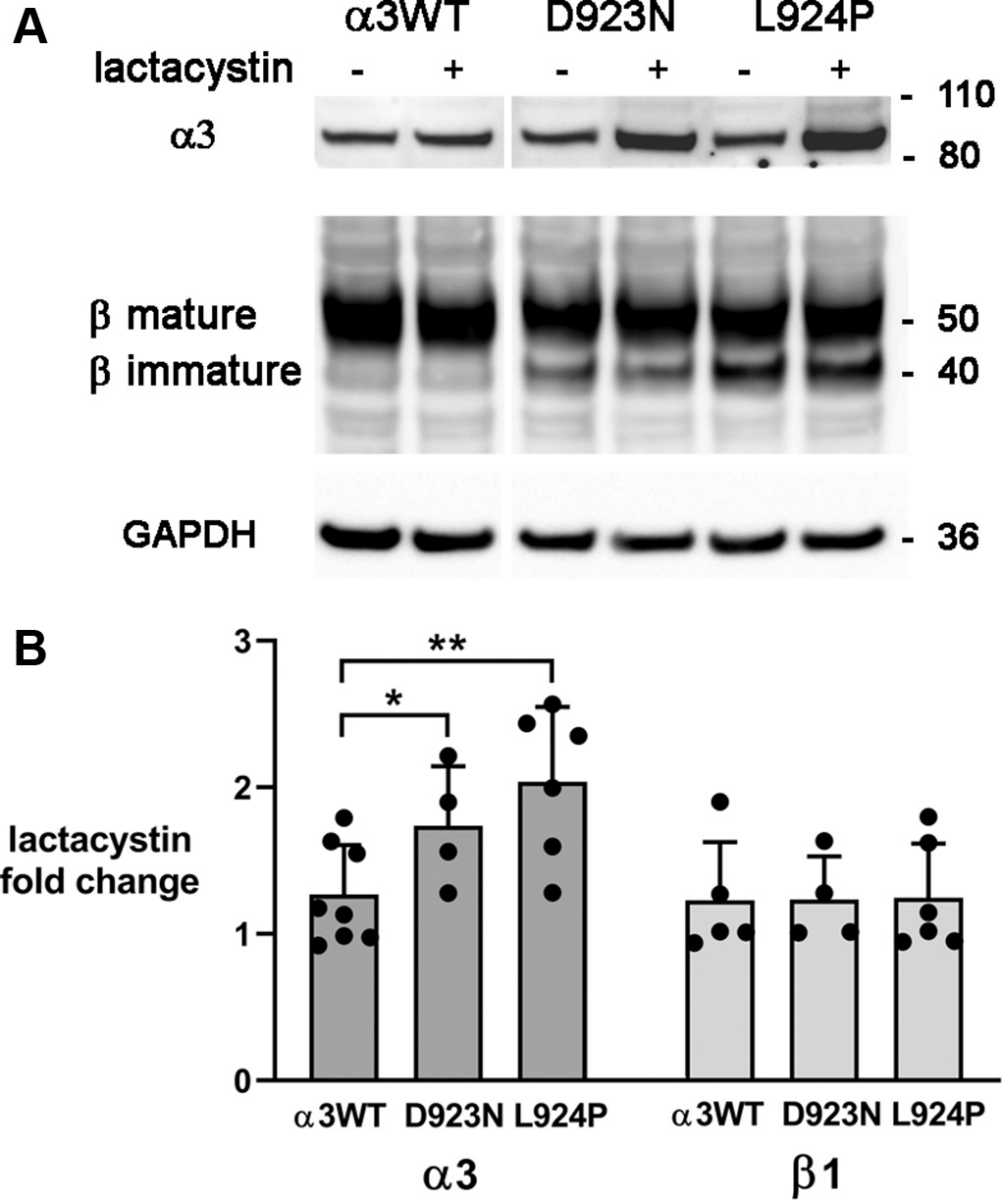
**Evidence for proteasomal degradation of both mutations.***A*, representative blot of α3 and β1 in cultures induced chronically with tet and then treated or not for 16 h with the proteasome inhibitor lactacystin. All pieces were from the same blot. *B*, quantification of n = 8 for α3WT, n = 4 for D923N, and n = 6 for L924P. ∗∗*p* < 0.001. It is notable that protease inhibition did not affect either the amount of β1 or the proportion of its immature form.

A constitutive method to dispose of misfolded ER proteins is ER-associated degradation (ERAD). Chaperones identify and help extrude the misfolded protein to cytoplasm. In the cytoplasm the misfolded protein is ubiquitinated and digested by the proteasome (43). Blocking the proteasome with lactacystin prevents digestion and raises the level of the misfolded protein. Figure 5 shows this for D923N and L924P compared with α3WT. The baseline level of each protein was set to 1. There was some (∼20%) rescue of the α3WT subunit. This is not surprising because, although the tet-induced α3WT construct is ostensibly present in only one copy in the DNA, its expression is under an exogenous promoter and there may be an excess of copies transcribed and translated. The extra α3 may be in the cytoplasm and thus still be nonfunctional. Both mutations showed elevated levels, greater and statistically significant for L924P. Importantly, the level of α3 was increased, whereas that of β1 showed no significant change. There was also no change in the proportion of immature to mature β1. This is consistent with the premise that complexation of α3 with β1 promotes association with chaperones like calnexin and results in α3 retention rather than being routed to ERAD. The data appear to rule out the possibility that a complex of mutant α3 and endogenous β are degraded by ERAD together.

**Figure 6:**
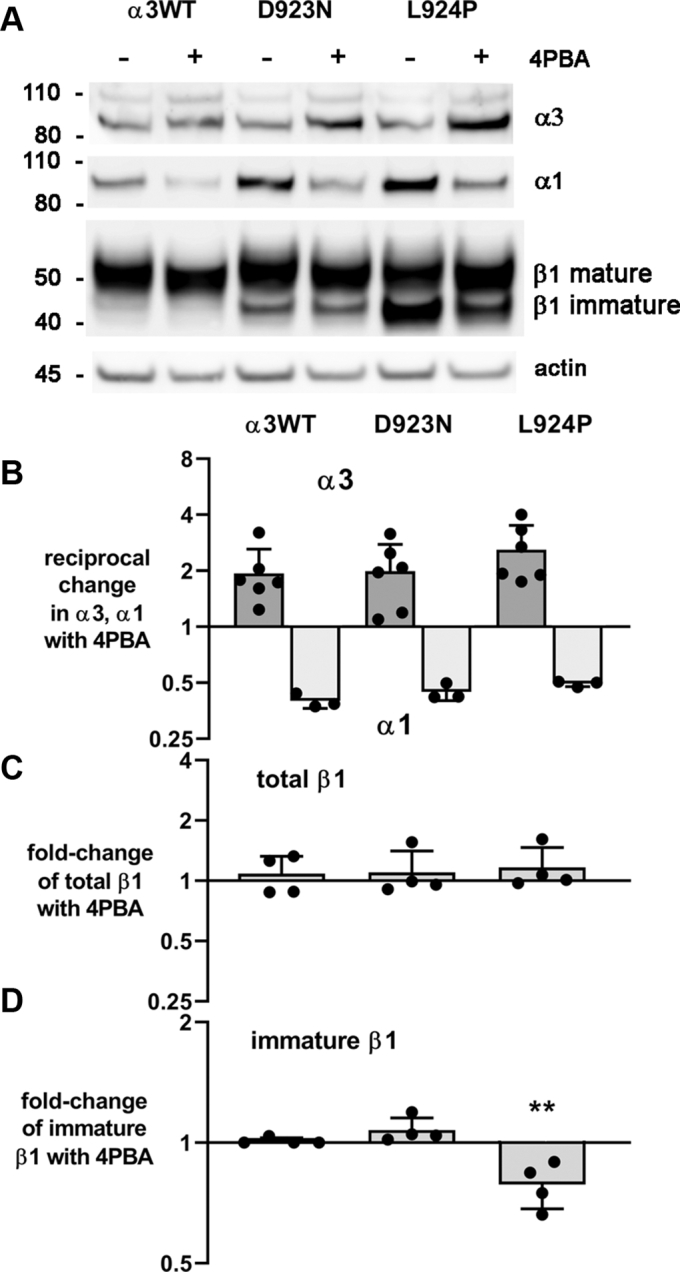
**A pharmaceutical corrector rescued expression of α3 and enhanced its trafficking out of the endoplasmic reticulum.***A*, replicate representative blots from one experiment were cut and stained with different antibodies. Incubation in 4PBA for 48 h resulted in a relative increase in α3 and decrease in α1; no change in the total amount of β1; but a shift of β1 in L924P-expressing cells from the immature to the mature glycosylated form, a marker of transfer from endoplasmic reticulum to Golgi. *B*–*D*, the results were quantified for n = 3 or 4 independent experiments. Scans of lanes were individually normalized to actin or GAPDH. *B*, reciprocal changes in α3 and α1, normalized to the control untreated sample in each experiment. The reciprocal change in expression is consistent with increased competition of the exogenous α3 for a rate-limiting factor, such as β subunit. *C*, expression of β1 appeared to be increased slightly but not significantly by 4PBA in all cell lines. *D*, the proportions of immature β1 were the same as seen in Figure 1 in the three cell lines. For L924P only, there was a statistically significant reduction in the immature form, *p* < 0.01, with a shift to the mature form.

Since the proteasome inhibitor showed that there was ongoing degradation of mutant α3 during biosynthesis, we tested the hypothesis that treatment of the cells with a chemical chaperone (a corrector) would improve both the recovery of α3 and the maturation of β1. Cultures grown chronically in tet were incubated with or without 4-phenylbutyrate (4PBA) for 48 h. 4PBA has two types of activity pertinent to protein biosynthesis: it has been shown in many cases to promote the folding of mutated membrane proteins, but it is also a histone deacetylase inhibitor with a broad stimulatory effect on transcription by loosening the interaction of histones with DNA. Evidence for both activities was seen (Fig. 6*A*). There was an increase in expression of α3 in α3WT-, D923N-, and L924P-expressing cells (Fig. 6*A*). A critical indication that 4PBA not only improved α3 expression but that the effect was likely to be due to better folding is that there was an accompanying decrease in endogenous α1 (Fig. 6*B*). As we showed earlier using acute tet induction, exogenous wildtype α3 and endogenous α1 competed for expression, whereas mutant L924P α3 was poorly competitive (15). Here 4PBA treatment increased the competitiveness of L924P up to the same level as α3WT, roughly normalizing it (Fig. 6*B*). In parallel, in all the cell lines the increase in α3 and decrease in α1 occurred without a major change in total β1 subunit (Fig. 6*C*). Notably, however, there was a shift of immature β1 subunit to the mature form for L924P (Fig. 6, *A* and *D*). Because immature β is characteristic of ER alone, its reduction is evidence that 4PBA treatment promoted passage of the αβ complex to Golgi and probably to plasma membrane.

**Figure 7:**
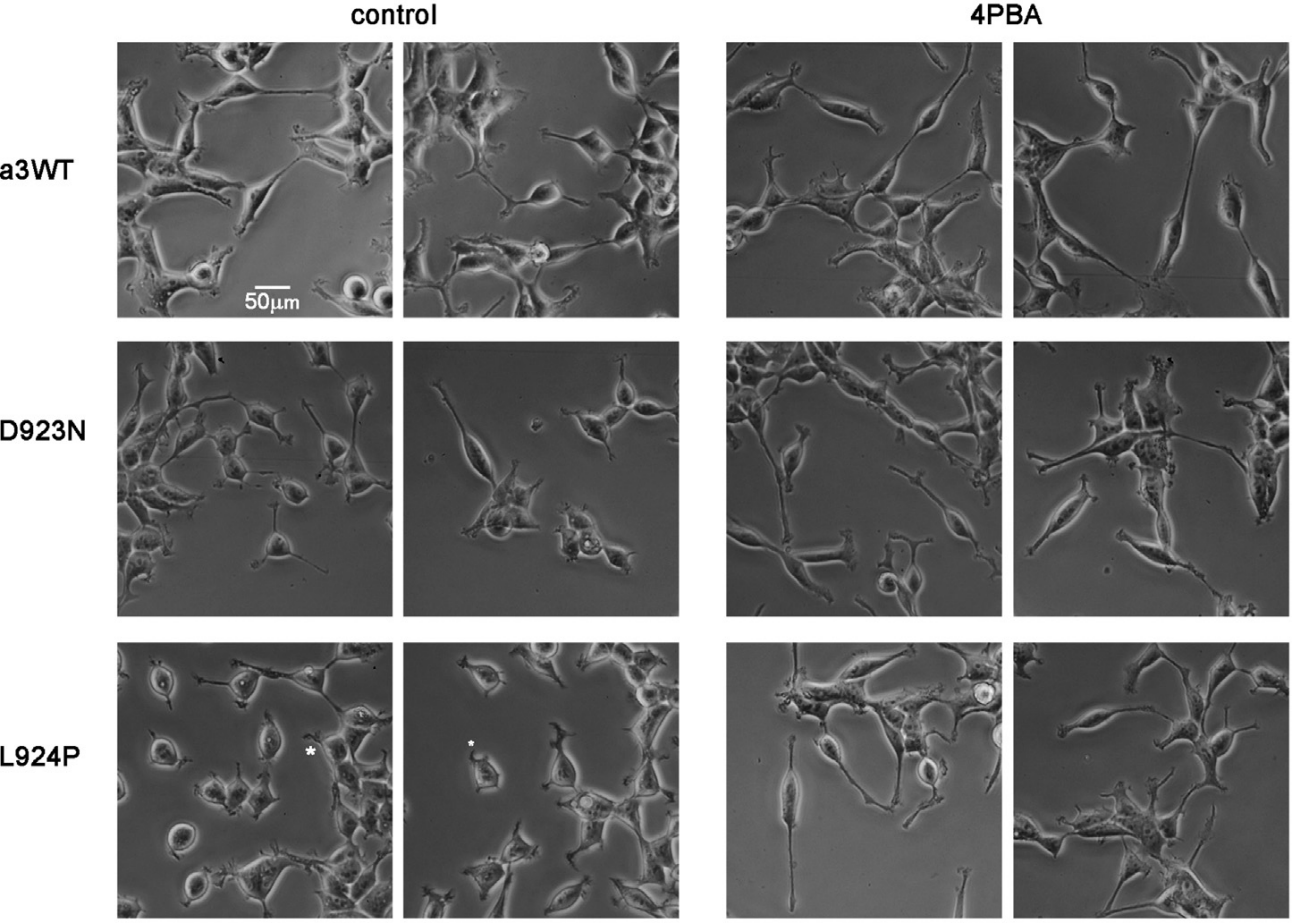
**4-Phenylbutyrate (4PBA) treatment improved the morphology of L924P-expressing cells.** Chronically induced cells were plated at relatively low density in chamber slides so that morphology could be observed. Fresh medium with or without 4PBA was added 24 h after replating. Phase-contrast pictures were taken after an additional 24 h. Asterisks (∗) in L924P cells without 4PBA treatment show stubby pseudopod extension still present 48 h after replating. Representative of four independent experiments. The scale bar represents 50 μm.

Treatment with 4PBA also affected the microscopic appearance of the mutant isogenic cell lines (Fig. 7). In previous work, we showed immunofluorescence images of cells that were mounding on top of each other, stained with antibodies for α3 and β1. The α3WT cells formed polygonal interfaces with each other, indicating tight apposition. D923N cells showed some polygonal interfaces, and also aggregations of α3 immunofluorescence. L924P cells, on the other hand, had even more aggregates; intracellular as well as surface β1; and cell–cell contacts that were not tight and symmetrical (15). Here phase contrast microscopy was used to visualize morphology and the processes extended by the cells when grown at lower density. α3WT cells showed no major change after 48 h of 4PBA administration. L924P cells, on the other hand, were less clustered and had impaired process extension in the untreated cultures. Some untreated L924P cells clustered normally, but there were many more unclustered cells. During 4PBA treatment, the cells acquired the same morphology as α3WT cells. This paralleled the shift of β1 subunit from the immature to mature glycosylated form seen in Figure 6.

**Figure 8:**
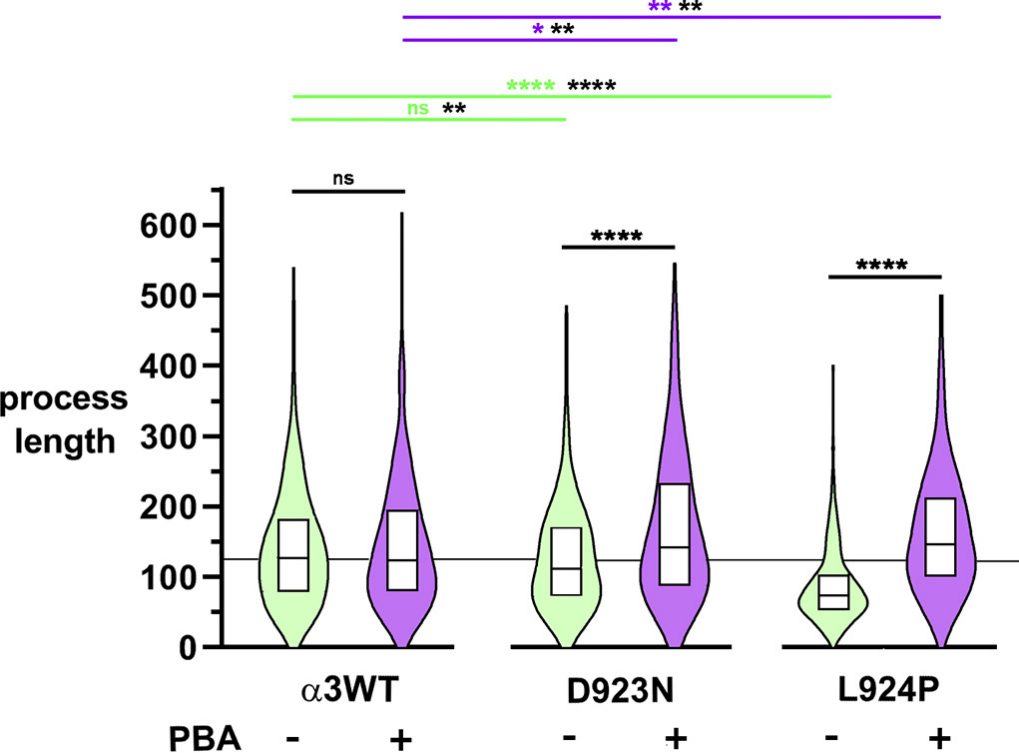
**Combination box and kernel density plots show the effect of mutation and of 4-phenylbutyrate (4PBA) on the length of cell processes.** Units of length are arbitrary as reported by ImageJ but rarely exceed 2 cell body lengths. The midline of each box is the median length, and the top and bottom edges of the boxes are the quartiles of distribution. Median lengths were reduced 12% in D923N cells and 42% in L924P cells. 4PBA had little effect in α3WT (n > 300 per condition) but more than normalized the measurements in both mutations (n > 200 per condition). The data are representative of one of four independent experiments. The tips of the violin plots were truncated at the longest measured length in each group. Data were analyzed by Mann–Whitney tests of significance with Tukey’s analysis for multiple comparisons. The comparisons in black show the improvement in process length with 4PBA treatment. The comparisons in green and magenta show the significance of differences between α3WT and each mutation. Additional comparisons with *black asterisks* are one-tailed tests to increase sensitivity to reductions and increases in the medians, which was important to detect the significance of the reduction of length in untreated D923N. ∗, <0.05. ∗∗, <0.01. ∗∗∗∗, <0.0001.

Quantification of process length in 200 to 300 cells per condition showed the differences in median lengths and quartile distributions with and without 4PBA (Fig. 8). See the figure and legend for statistical analysis. Median lengths were the same in α3WT with and without 4PBA. In untreated D923N, lengths were intermediate between α3WT and L924P cells. In both mutants, lengths were corrected by 4PBA. In the untreated cultures, D923N median length was 12% lower than α3WT, and L924P was 42% lower. In the treated cultures, there was a 26% increase for D923N and a 97% increase for L924P. Both mutant cell lines were fully restored by this criterion.

It should be noted that the microscopy experiments were performed without ouabain, so endogenous α1 was active and the cells were not dependent on residual α3 activity. This is evidence that the aberrant morphology is unlikely to be a consequence of ion transport limitation but instead is a cell-intrinsic consequence of mutation that can be ameliorated by 4PBA.

## Discussion

### Mechanistic and cellular aspects

If reduction or inactivation of pump activity were the only consequence of *ATP1A3* mutation it would be difficult to understand why some mutations do not manifest until adulthood, whereas others produce symptoms at birth. Our previous work showed that cells expressing either D923N or L924P had enough α3 Na,K-ATPase activity to support the cell growth of stable, isogenic transfectants when endogenous α1 was inhibited (15). D923N was previously shown to have significantly reduced affinity for Na^+^ (44). Here, the measured ATPase activity in enriched plasma membrane fractions of the two mutations was very similar and a fraction of that of WT. There were major differences observed in the degree of activation of the UPR between the two mutations, however. First, there were differences between D923N and L924P in ER retention of the β1 subunit, and sucrose density gradient fractionation showed a parallel effect on α3 subunit ER retention. Second, the increased level of α3 detected in ER-enriched fractions was itself an underestimate because inhibition of ERAD by the proteasome inhibitor lactacystin showed that some mutant α3 was being degraded, much more for L924P than for D923N. There was no evidence that β1 was being degraded by ERAD, so degradation of misfolded α3 may have preceded αβ complex formation. These adaptations (ER retention and ERAD) at the earliest stage of biosynthesis were paralleled by ongoing activation of a central event in UPR defensive mechanisms: inhibition of eIF2α by its phosphorylation by the UPR receptor PERK, which reduces the load on the ER by broadly reducing mRNA translation (37). Complementary to reducing protein translation, the UPR typically causes expansion of the ER. This is consistent with the observation that the ER-enriched sucrose gradient fractions had increasingly larger fractions of total protein: α3WT < D923N < L924P. In terms of Na,K-ATPase protein structure, a difference in misfolding during biosynthesis makes sense because D923 contributes to the third Na^+^-binding site on the ion-binding side of the M8 transmembrane helix, where the polar, similar-sized asparagine substitution may cause more functional than structural damage, whereas introducing a kink with a proline substitution for L924 is potentially structurally disruptive to M8 itself, to the proper positioning of D923, to the beginning of the intracellular L8-9 loop, and to M8’s interactions with M5, M6, and M9 (illustration in (15)).

Activation of the UPR is likely to enhance the pathogenicity of *ATP1A3* mutations that produce early onset of symptoms in patients. Its defensive arm, adaptation to misfolding, was successful in the stable lines expressing these two particular mutations: the cells survived and divided indefinitely, making it possible to do a direct comparison of their UPR responses as well as to fractionate membranes. Apoptotic signaling nonetheless was present. BAD, a BCL-2 family member, has up to 10 phosphorylation sites targeted by different kinases, making it a hub for incoming signals (39). BAD has a pivotal role in regulating apoptosis in the UPR by promoting either adaptation (when phosphorylated) or apoptosis (when dephosphorylated) through different partners (38). Apoptosis is activated when adaptive UPR responses are insufficient to prevent the toxic accumulation of misfolded protein (17). A reduction in phosphorylation of BAD at Ser99 (also known as Ser136 from work in mice) is permissive for the UPR to proceed to apoptosis (38). BAD’s dephosphorylation here shows sensitization to apoptosis of the cells expressing either mutation. HEK-293 is disadvantageous for investigation of apoptosis itself because it has an adenoviral genome fragment, essential for indefinite proliferation, that encodes oncogenes E1A and E1B, and E1B acts to inhibit apoptosis pathways (45). How other pathways associated with the UPR contribute to apoptosis remains to be investigated, preferably in cells that can be differentiated into neurons that normally express α3. Alterations in proteostasis affect synaptic plasticity and are implicated in a number of cognitive disorders, neurodegenerative diseases, microcephaly, and atrophy (46, 47).

### Impacts of subunit interaction and competition

The existence of competition of α subunits during biosynthesis complicates the interpretation of Na,K-ATPase mutations. During acute induction of exogenous α3WT, the level of α1 fell, whereas the total was constant as shown with a pan-specific antibody (15). Whether the limiting factor during biosynthesis is β subunit itself or some other essential cofactor (48), the total of α was operationally capped. Competition is similarly expected between WT and mutant α3 alleles. In the circumstance of no misfolding, the final levels of WT and mutant alleles of α3 should be 50:50. When one allele of α3 misfolds, its final proportion should be less than 50%. That would appear to be a better outcome (a lower proportion of mutant protein), whether the mutation caused loss of function or a toxic gain of function, if it were not for the major cell biological effects of the UPR.

Fractionation of membranes by density has not been utilized in the literature on the UPR response to misfolded proteins, but it revealed some interesting remodeling features here. First, it produced direct evidence of retention of α3 in RER. Second, for both mutants, some immature β was found in the lightest gradient fraction. We had previously showed than no immature β1 was accessible to biotinylation from the extracellular surface of the cells (15), but the light membranes were enriched in SER and Golgi as well as plasma membrane. Assuming the immature β1 is in ER, its low density indicates that it is likely to be devoid of ribosomes, *i.e.*, SER. Third, a comparison of the distributions of α3 and α1 on the sucrose gradients shows that in D923N cells, α3 and α1 distributions were indistinguishable and similar to α3WT, whereas in L924P cells, distributions of α3 and α1 were also indistinguishable but very different from that seen in α3WT and D923N. The distribution of total β1 subunit was similarly altered in L924P, and intermediate between α3WT and L924P for D923N. In principle, this pleiotropic effect on wildtype α1 could reflect a classic dominant negative effect mediated by heterodimerization of α3 and α1, but we are aware of no evidence that this ever happens. In the older literature, dimerization or tetramerization of α1 was supported by much indirect evidence (49) and also by coprecipitation of two tagged α1 constructs expressed in insect cells (50), but dimerization has not been supported by any analysis of structure and may involve scaffolding intermediaries (51) or adventitious association in detergent (52, 53). Instead, we speculate that the impact of L924P α3 on α1 membrane distribution is due to the extent of membrane remodeling, and that it is a UPR effect with potential to be deleterious. It remains to be seen if other membrane proteins are affected in parallel. With several possible effects interacting dynamically, the similar gradient distributions of α3, α1, and β1 in L924P cells need additional study. It is also likely that some labile Na,K-ATPase mutations will have accelerated internalization from the plasma membrane and lysosomal degradation, an aspect beyond the scope of this study.

Notably, the improved folding of α3 in 4PBA was accompanied by a reduction of α1, implying that correction of misfolding of α3 leads to improved α3-α1 competition. The positive response to 4PBA holds out hope for enhancement of protein folding as a therapeutic approach for some mutations (*i.e.*, when the well-folded mutant has some activity but does not have a toxic gain of function like an ion leak). 4PBA has been used experimentally to improve expression of misfolding mutations in other P-type ATPases in culture (ATP12A, ATP7B, ATP8B1) (54, 55, 56). It was effective in both D923N-and L924P-expressing cells but had little effect in α3WT-expressing cells. Remarkably, in L924P cells it improved the maturation of the β subunit and normalized process extension by the cells, possibly owing to improved substrate adhesion because β has an additional role as an adhesion molecule (57, 58). It did this without a change in total expression of the β subunit, consistent with an improvement in cell surface expression. Whether 4PBA’s activity as a histone deacetylase inhibitor had a beneficial effect is not known. 4PBA itself is an US Food and Drug Administration–approved drug (Buphenyl), but this is because it is metabolized and eliminated rapidly (https://www.accessdata.fda.gov/drugsatfda_docs/label/2009/020572s016,020573s015lbl.pdf; April 13, 2020) and its metabolic product, phenylacetate, conjugates with glutamine and improves excretion of nitrogen in patients with urea cycle disorders. Small molecule misfolding correctors with appropriate pharmacodynamics (59) or strategies to activate folding pathways (60) may be effective for *ATP1A3* disease.

In conclusion, despite the similarity of having enough residual activity to support the growth of cells, these two *ATP1A3* mutations produced different human phenotypes that correlated here with the magnitude of ER retention, eIF2α inhibition, and lactacystin response, and with the redistribution of membranes on sucrose density gradients. The greater severity of the cell biological disruption in the mutation with the more severe phenotype, L924P, suggests that misfolding during biosynthesis contributes to pathogenicity separately from functional impairment of the enzyme (12, 44).

## Experimental procedures

### Isogenic cell lines

Stable lines expressing ATP1A3 and the mutants D923N and L924P were generated in HEK Flp-In T-REx^TM^ 293 cells as described previously (15). Cells were grown in DME medium (Cellgro) containing 4.5 g/l glucose, 2 mM L-glutamine, 100 units/ml penicillin/streptomycin mixture, and 10% fetal bovine serum (tet-free) (Omega Scientific, Inc, Tarzana, CA, USA) in the presence of blasticidin S and hygromycin (InVivogen, San Diego, CA, USA). Induction of wildtype and mutant ATP1A3 was with tetracycline (Sigma-Aldrich, Inc). Where indicated, cultures were grown in 3 μM ouabain to force them to be dependent on exogenous α3.

### Western blot analysis

Cell lysates were produced with RIPA buffer containing 20 mM Tris-HCl, pH 7.5, 150 mM NaCl, 1 mM Na-EDTA, 1 mM Na-EGTA, 1% NP-40, 1% sodium deoxycholate, supplemented with protease inhibitor cocktail (Complete Mini, Roche Diagnostics, Mannheim, Germany) and phosphatase inhibitor cocktail 3 (MilliporeSigma). Sample preparation for gels was described elsewhere (15). Gel electrophoresis was in Nu-Page 4% to 12% gradient Bis-Tris NuPAGE gels (Invitrogen). Proteins were transferred to nitrocellulose and stained with various antibodies. α3 Subunit of Na,K-ATPase was detected with the goat peptide-specific polyclonal antibody C16, or with the mouse monoclonal antibodies, clones H4 or F1 (all from Santa Cruz Biotechnology). Monoclonal antibodies 6F (Developmental Studies Hybridoma Bank, University of Iowa) or M17-P5-F11 (gift of Dr James Ball, University of Cincinnati (61)) were used to detect human α1 and β1 subunits of Na,K-ATPase, respectively. Antibodies against eIF2α (5324), phospho-eIF2α (9721), and phospho-BAD (4366) were from Cell Signaling Technologies (Danvers, MA, USA). Actin–horseradish peroxidase (HRP) and GAPDH-HRP antibodies were from Santa Cruz Biotechnology. For Western blot analysis, secondary antibodies were HRP-conjugated, and final detection was with chemiluminescence (WesternBright ECL, Advansta, Menlo Park, CA, USA). An LAS 4000 imager (GE Healthcare Life Sciences) with ImageQuant TL software was used to quantify Western blots. A minimum of three biological replicates were used for analysis. Loading control was with quantification of actin or GAPDH.

Expression levels of α3WT, D923N, and L924P are generally comparable but not identical between experiments. It is worth mentioning that, when comparing the unfolded protein response between Na,K-ATPase mutations, normalization of any data to equal protein expression levels of mutant and WT α is not desirable because too many factors affect the outcome. First, α1 and α3 compete, and misfolded protein does not compete as well (15). Second, expression encompasses transcription and translation, but it is also impacted by the UPR posttranslationally: regulation of ERAD and autophagy tend to decrease protein and ER retention to increase it. Furthermore, mutations differ in how much gets to the membrane and how much accumulates in aggregates (15), which could affect the interpretation of immunoblot signals. Expression is even potentially regulated by mRNA degradation *via* the endonuclease activity of IRE1 (RIDD, regulated IRE1-dependent decay of mRNA), or miRNA suppression of translation activated by the UPR, even for exogenous transcripts. Furthermore, α and β fates interact, but Figures 2, 5, and 6 show that they are not always proportional.

### Fractionation of cell membranes

Isogenic cell lines expressing WT, L924P, or D923N ATP1A3 were grown until confluent in a medium supplemented with 3 μΜ ouabain. Cells were scraped in 250 mM sucrose in buffer A (10 mM Tris-HCl, 2 mM EDTA, pH 7.4) containing protease inhibitor cocktail (Roche Diagnostics). Disruption was with a tight Dounce homogenizer followed by centrifugation for 15 min at 500*g* and 4 °C. The supernatants were combined with 2.55 M sucrose in buffer A in 1:1 proportion (v/v). The membrane mixture was fractionated on a sucrose density gradient composed of 2, 1.6, 1.2, and 0.8 M step sucrose solutions in buffer A (details in (62)). The sample mixture (1.4 M sucrose in buffer A) was layered between the 1.6 and 1.2 M layers. Centrifugation was in an SW28.1 rotor, for 6 h at 25,000 rpm, 4 °C, in a Sorvall Discovery 90E centrifuge. Fractions enriched in plasma membranes, Golgi, and endoplasmic reticulum were collected at approximately 1, 1.3, and 1.5 M densities, respectively. The final precipitation of membranes was achieved by dilution with a buffer containing 25 mM imidazole and 2 mM EDTA, pH 7.4, and centrifugation in a type 45 Ti rotor at 33,000 rpm for 1 h, 4 °C. The pellets were resuspended in 250 mM sucrose, 1 mM EDTA, 10 mM Tris pH 7.4 and stored at −70 °C. Final sample volumes and protein concentrations were determined to calculate yield.

Equal amounts of proteins were separated in SDS gels, the blots were scanned, and the proportions of α3, α1, and β1 subunits were calculated for different fractions of the gradients. Comparisons of enrichment were made only within blots, but to average the results of multiple independent experiments with different antibody exposure intensities, the sum of the antibody signals from 10 μg of protein from dense (D), intermediate (I), and light (L) gradient fractions were normalized to 1.0 for α3, α1, and total β1 (mature + immature). The same normalization was applied to immature β1 from L924P cells, but the levels of immature β1 from α3WT and D923N are shown as their actual proportion to L924P. Comparisons of distribution were made by calculating the proportion of the total in each fraction.

### ATPase assay

ATP hydrolysis was measured in plasma membrane gradient fractions by the basic Fiske-Subbarow method described at length elsewhere (33). The buffered SDS method described in the same protocol paper did not improve results for α3WT or D923N membranes and inactivated L924P.

### Activation of the unfolded protein response

qPCR was used to detect the spliced form of XBP1 mRNA, XBP1s. Total RNA from 10^6^ cells from a3WT, L924P, or D923N cells grown in BHT medium with or without 3 μM ouabain was isolated with RNeasy kits (Qiagen). Single-strand DNA was synthesized with the iScript Advanced cDNA kit (Bio Rad). The primers were the following: Xbp1S forward, AACCAGGAGTTAAGACAGCGCTT; Xbp1S reverse, CTGCACCTGCTGCGGACT. qPCR analysis was performed on the Applied Biosystems StepOnePlus Real-Time PCR System. Actin mRNA was used as the calibrator.

### Rescue of misfolded mutants

For inhibition of proteasome degradation, cells were grown in 12-well plates in medium supplemented with blasticidin, hygromycin, and tetracycline antibiotics (DME-BHT medium). Proteasome inhibitor lactacystin (Alexis Corp, Switzerland) was dissolved in water (1 mM stock solution) and applied to cells at 5 μM overnight in the incubator.

For treatment with a pharmacological corrector/chemical chaperone, cells were plated at 30% to 40% density in BHT medium in 12-well plates. Twenty-four hours later, sodium 4-phenylbutyrate (EMD Millipore Corp) was added to final concentration of 5 mM, and the treatment continued for 48 to 72 h. Cell lysates with RIPA buffer were used for Western blot analysis. In parallel, cells were plated at 10% to 15% density in eight-chamber slides (Nunc) and treated as above. Phase contrast pictures were taken at 24, 48, and 72 h.

## Data availability

All the data are contained within the article.

## Conflict of interest

The authors declare that they have no conflicts of interest with the contents of this article.
